# Reviews: Rapid! Rapid! Rapid! …and systematic

**DOI:** 10.1186/2046-4053-4-4

**Published:** 2015-01-14

**Authors:** Holger J Schünemann, Lorenzo Moja

**Affiliations:** Department of Clinical Epidemiology and Biostatistics, McMaster University, HSC Room 2C16, 1280 Main Street West Hamilton, Hamilton, ON L8S 4K1 Canada; McMaster GRADE Centre, Hamilton, Canada; Cochrane Applicability and Recommendations Methods Group, McMaster University, Hamilton, Canada; Department of Biomedical Science for Health, University of Milan, Milan, Italy; Clinical Epidemiology Unit, IRCCS Galeazzi Orthopedic Institute, Milan, Italy

**“Although time is a reality, lack of time is not lack of reality” - HOJES**

## Systematic reviews

Systematic reviews are scientific investigations, with pre-planned methods and an assembly of original studies as their “subjects” [1, 2]. They synthesize the results of multiple primary investigations by using strategies that limit bias and random error [1, 2]. Systematic reviews are transparent about how studies were identified and which were included or excluded, the risk of bias assessment, and the methods to summarize data and assess the certainty in the evidence. Standards for the conduct of systematic reviews have been made available by the Cochrane Collaboration and other organizations. If systematic reviews are done well, e.g., by adhering to conduct (e.g., Cochrane Handbook for Systematic Reviews of Interventions) and reporting (e.g., PRISMA Statement) best practice standards, it is not sensible to question the value of systematic reviews as a source of information for shaping decision making [3, 4]. This methodology of systematic reviews—although laid out three or more decades ago—is continuously and rapidly updated by scientists specializing in research synthesis. Now, *Systematic Reviews* is publishing a series of articles including methods and examples of accelerating approaches to conducting literature reviews. As a rule of thumb, rapid systematic reviews should be conducted in less than 8 weeks, including protocol publication. On the whole, this is a saving of about 75% in terms of time compared to what most researchers would propose as standard timeline for systematic reviews. Examples will highlight how health policy decisions can be influenced when a rapid review methodology is used.

## The challenge of traditional systematic reviews

Findings from a single randomized trial are often rapidly challenged by succeeding studies, and rigorous systematic reviews help approximate “true evidence” and estimates in effects [5]. High-quality systematic reviews are used more often and are considered more trustworthy by health professionals in terms of relevance to clinical practice than other types of designs [6]. However, conducting and adhering to the standards of traditional systematic reviews can be time consuming. The reason for that lies in the rigorous approach to methods ensuring that the best available evidence is identified, assessed, and synthesized. But those demanding evidence syntheses for decision making are increasingly living in faster paced times, influenced by innovative interventions and technology that accelerate communication and interaction. Decision makers often do not appreciate the intricacies of research methods and the time needed to comply with the task. The argument that transparency is ensured by completing a traditional systematic reviews is often not convincing enough. But there are other reasons.

## The rationale for rapid—systematic—reviews

The concern regarding a timely decision on health care and policies is the driving force for rapid reviews. In fact, decision making should not be delayed in most situations and cannot be delayed in some. In the face of a tragic Ebola epidemic, we are reminded of how rapidly answers are required. To base answers on the best available evidence, this evidence must be synthesized without undue delays. While typical systematic reviews can take years to complete (one of the author was involved in a systematic review that took 12 years to complete), rapid reviews are required when facing such dramatic situations. Prior to the Ebola epidemic, the fear of avian influenza prompted the World Health Organization to offer rapid guidelines that were supported by a rapid review methodology. From guideline panel formation to completion of the recommendations, only about 12 weeks passed [7]. Another recent rapid systematic review was commissioned to inform decision making with regard to the safety of two drugs, bevacizumab (Avastin) and ranibizumab (Lucentis), widely used to stabilize vision in patients with neovascular age-related macular degeneration. From team formation to completion of the systematic review, only about 8 weeks passed. Another 8 weeks was necessary to complete the publication process and publish it as a Cochrane review [8]. These and other experiences, such as the ones presented in this series, show that rapid evidence synthesis can be done to support decisions ranging from clinical to health policy.

## The pitfalls of rapid reviews

Should rapidity be considered as a key risk factor for poor, overly simplistic, or frankly misconducted systematic reviews? We do not think so. Rapidity by itself is not a predictor of the quality of a systematic review. The same amount and quality of work can be completed in a shorter or longer time, although sometimes saving in time might be accompanied by compromising in conduct. This includes missing important evidence and errors in the assessment or synthesis of the evidence. Systematic review authors and users of systematic reviews must, however, resist the pressures of shortcuts when they suggest bias [9]. The quality of systematic reviews and meta-analyses should be evaluated irrespective of their speed.

## What rapid—systematic—reviews must do

Apart from time, what makes rapid systematic reviews different from traditional systematic reviews? Not the amount of work. Rapid reviews must remain systematic by adhering to the core principles of systematic reviews that avoid bias in the inclusions, assessment, and synthesis of studies. The methods sections will be of greater importance as deviations from traditional systematic review methods should be laid out clearly. Thus, contrary to what the label “rapid” may imply, transparency in the description of the methods used will become more important; rapidity is not a justification for brevity, and rapidity should not be confused with brevity. One approach to increasing transparency will be highlighting where the PRISMA criteria were omitted or modified. Rapid reviews can remain systematic if the core principles are adhered to, and that should be reflected in the methods and title.

## A note on resources

An important issue that might differentiate rapid systematic reviews from traditional systematic reviews is the more marked need to support production across the review’s lifecycle, from early question generation and method planning to development of the manuscript, followed by the release of user-friendly communication tools (e.g., summary of finding tables). The speed of review conduct can be directly correlated with the availability of resources, both human and financial (which, in turn, may ensure human resources). Adequate planning requires lining up all activities against review deliverables and timelines and harmonizing the required expertise in a more streamlined fashion. Since rapid systematic reviews often require reaching a consensus about disputed evidence more quickly, they might involve stakeholders with different backgrounds earlier. This aspect also has resource implications, since review drafts will circulate quickly between authors and require closer attention to each round of revision. When rapid reviews include many studies, it might be expedient to increase the number of reviewers involved. However, increasing the team size has costs, too: the possibility of greater interindividual reliability on study inclusion and data abstraction must be accepted. It is important to ensure that all reviewers are well trained in systematic review methods and ensure attention to review execution at each step even under pressure. In this way, producing rapid systematic reviews that do not fall short in terms of the applied methodological rigor remains a reality.

## Summary and terminology

In summary, if there is no compromise of the validity of a review, then reviews should be done rapidly. This would mean that one does not accept shortcuts in terms of methods for review conduct. Thus, the term rapid review is a possible misnomer and conceptually wrong (in the authors’ view). Velocity does not have to impact transparency and appropriate methods. Rapid reviewers must do their utmost to adhere to guidelines for review conduct and reporting. As evidence is only slowly emerging as to which steps in the systematic review process may be altered by increased speed and will require more examples such as the ones described by the authors of this series in *Systematic Reviews*, transparency is key. Rapid reviews that are not systematic bear the risks of any other narrative review or poorly conducted systematic reviews [10].
